# Detection of *Klebsiella pneumoniae* in Veterinary and Food Matrices Using Loop-Mediated Isothermal Amplification

**DOI:** 10.3390/pathogens14030296

**Published:** 2025-03-19

**Authors:** Icía Bermúdez-Fornos, Alberto Cepeda, Alejandro Garrido-Maestu, Alexandre Lamas

**Affiliations:** 1 Food Hygiene, Inspection and Control Laboratory (LHICA), Department of Analytical Chemistry, Nutrition and Bromatology, Veterinary School, Campus Terra, Universidade de Santiago de Compostela, 27002 Lugo, Spain; icia.bermudez@rai.usc.es (I.B.-F.); alberto.cepeda@usc.es (A.C.); 2 Laboratory of Microbiology and Technology of Marine Products (MicroTEC), Instituto de Investigaciones Marinas (IIM), Consejo Superior de Investigaciones Científicas, Eduardo Cabello, 6, 36208 Vigo, Spain

**Keywords:** loop-mediated isothermal amplification, *Klebsiella pneumoniae*, naked eye, urine, blood, milk

## Abstract

*Klebsiella pneumoniae* is an opportunistic human pathogen of high relevance due to its ability to acquire antibiotic resistance. This pathogen is included, along with *Enterococcus faecium*, *Staphylococcus aureus*, *Acinetobacter baumanii*, *Pseudomonas aeruginosa*, and *Enterobacter* spp., in the ESKAPE group, which consists of the most important bacterial pathogens resistant to antibiotics in clinical setups. Due to the importance of the rapid identification of infection-causative agents, a novel method for the rapid identification of *K. pneumoniae* was developed in the present work. This novel method was based on loop-mediated isothermal amplification (LAMP) and evaluated in real-time LAMP, as well as in end-point colorimetric LAMP. Additionally, the method was evaluated in two different clinical samples, namely, blood and urine, along with a food sample, namely, milk; four DNA purification protocols were also evaluated (thermal lysis, chelex, magnetic beads, and glass milk). The results revealed differences in the performance of the LAMP assays depending on the specific combination of the matrix–DNA purification protocol. Overall, the protocol reporting the best results in all the matrices was the one based on chelex, with which it was possible to reach an LOD50 below 10 CFU/mL after a short pre-enrichment step of 6 h in TSB. The method demonstrated reliability, sensitivity, and simplicity and could be performed by non-trained personnel thanks to the colorimetric format.

## 1. Introduction

Antibiotic resistance has been called “the silent pandemic” due to its direct and indirect implications on human health [1]. The most important bacterial pathogens associated with antibiotic resistance are *Enterococcus faecium*, *Staphylococcus aureus*, *Klebsiella pneumoniae*, *Acinetobacter baumanii*, *Pseudomonas aeruginosa*, and *Enterobacter* spp., which are typically known as the ESKAPE group, a term coined by Louis B. Rice in 2008 [2]. Among these, *K. pneumoniae* is a Gram-negative, encapsulated, non-motile bacterium that is present in the environment and an opportunistic human pathogen [3]. Given the fact that certain strains are capable of causing different types of infections in healthy individuals, it has been subclassified into two major pathotypes, classic (cKP) and hypervirulent (hvKP) [4]. It is worth noting that the classical pathotype is resistant to ampicillin, carbenicillin, and ticarcillin due to the production of the penicillinase SHV-1, and hvKP bears additional virulence factors that allow it to invade different types of tissues [5,6].

The diagnosis of *K. pneumoniae* is based on the culture and isolation of the bacteria from infected samples and/or contaminated foods, followed by its biochemical identification [7,8]. This procedure is highly subjective with a long turnaround time, as this is a major issue, particularly when dealing with infected individuals [9]. The higher acceptance of molecular techniques, particularly those based on DNA amplification, has allowed us to overcome these limitations. Even though polymerase chain reaction (PCR) can be considered the gold standard, the emergence of isothermal techniques, mainly loop-mediated isothermal amplification (LAMP), has broadened the number of suitable techniques for this task. It is worth noting that LAMP presents several advantages of PCR that make it very attractive; to name a few, due to its isothermal nature, no complex equipment is needed, and it is compatible with a wide number of detection strategies of simple interpretation, along with its reported higher resistance to typical chemical/biochemical amplification inhibitory compounds [10,11,12].

Given the importance of *K. pneumoniae* for human health, the goal of the present study was to develop a LAMP-based assay for its rapid detection and evaluate its applicability in clinical and food matrices.

## 2. Materials and Methods

### 2.1. Bacterial Strains and Culture Preparation

*K. pneumoniae* WDCM 00192, purchased from the Spanish Type Culture Collection (CECT), was selected as the reference strain for the development and evaluation of the novel assay. Pure cultures were prepared in brain–heart infusion broth (BHI) (Merck, KGaA, Darmstadt, Germany) and incubated at 37 °C overnight. The fresh, pure culture was streaked onto nutrient agar (NA) (nutrient broth from VWR Chemicals EC with 15 g of agar–agar from VWR Chemicals, Barcelona, Spain) plates, and the plates were incubated under the same conditions described above. For inoculation experiments, a few colonies were resuspended in a 0.85% NaCl solution until a turbidity of 0.2 was reached, which was estimated to be 10^8^ CFU/mL. This value was photometrically determined (Photometer, DEN-600, Biosan, Riga, Latvia). Reference values of viable bacteria were determined by plating one hundred-fold serial dilutions of the suspension on NA, and the bacteria were incubated as previously described.

In addition to the reference strain, a panel of non-target bacteria was also included in the present study to evaluate the inclusivity and exclusivity of the newly designed LAMP assay. A complete list of these microorganisms is provided in Table 1. Fresh cultures of all these were prepared as detailed above, and the DNA was extracted using the thermal lysis protocol specified below in Materials and Methods Section 2.3.

The blood was defibrinated sheep blood (allMedia, ref: 3049, purchased from Cientisol, Santiago de Compostela, Spain). Regarding the urine samples, these were obtained from cows and were collected by a veterinarian on a dairy farm; the collection procedure consisted of placing an empty bottle under the cow and waiting for spontaneous cow urination. Lastly, as a food matrix, whole, UHT milk purchased from a local supplier was used.

### 2.2. Primer Design

Primer design was performed with Primer Explorer V5 (https://primerexplorer.jp/e/index.html, accessed on 12 March 2025) with the default design parameters. It was decided to target the *khe* gene, which encodes de hemolysin, taking the genome NC_016845.1 as a reference. This genome was retrieved from RefSeq, and the complete gene sequence was imported into Primer Explorer for the design (https://www.ncbi.nlm.nih.gov/refseq/, accessed on 12 March 2025).

### 2.3. Nucleic Acid Extraction

DNA extraction in spiked samples was performed after a short pre-enrichment of 6 h in TSB at 37 °C. The protocols compared consisted of thermal bacterial lysis, followed by different purification strategies. These were previously described by Regal et al. [13]. For pure cultures and spiked urine samples, 1 mL of a freshly prepared bacterial culture was centrifuged at 16,000× *g* for 2 min; then, the supernatant was discarded and used for thermal lysis (TL) along with the corresponding purification protocol. Briefly, in the TL, the pellet was resuspended in 100 µL of nuclease-free water, heated at 99 °C for 5 min, and centrifuged, as indicated above, to pellet cellular debris and recover the DNA; in the chelex (CH) purification, instead of water, a 6% (*w*/*v*) Chelex^®^ 100 suspension was used (Bio-Rad Laboratories, Inc., Hercules, CA, USA); the mixture was incubated at 56 °C for 15 min and then for 8 min at 99 °C, both steps under constant agitation. Then, the samples were centrifuged, as indicated above, to recover the supernatant with the DNA. For the magnetic bead purification (MB), 100 µL of Mag-Bind^®^ beads were added, incubated at room temperature for 5 min, and then separated with a magnet and rinsed twice with 70% ethanol; finally, the purified DNA was eluted in 100 µL of nuclease-free water. Lastly, the “glass milk” (GM) protocol consisted of resuspending the pellet in 100 µL of nuclease-free water and 100 µL of 4% SDS. This mixture was incubated at 99 °C for 5 min; then, 400 µL of isopropanol, 200 µL of NaCl, and 10 µL of “glass milk” were added, incubated for 5 min at room temperature, and centrifuged. The pellet was rinsed twice with 70% ethanol, the final pellet was heated for 5 min at 65 °C, and, finally, the DNA was eluted in 100 µL of nuclease-free water.

In the particular case of the blood samples, 900 µL of a sample was mixed with 100 µL of lysis solution (0.8 NH_4_Cl, Merck, KGaA, Darmstadt, Germany) and incubated at room temperature for 10 min. After incubation, the sample was centrifuged, as previously described, the supernatant was discarded, and the pellet was resuspended in 1 mL of PBS, pH 7.4 (Invitrogen, Thermo Fisher Scientific Inc., Waltham, MA, USA), and centrifuged again, as described above.

When dealing with the milk samples, after the initial centrifugation step, a fat layer was observed. This was removed with a spatula; then, the pellet was rinsed with 1 mL of PBS and centrifuged again, as described. All DNA extracts were stored either at 4 °C or −20 °C for short- and long-term storage, respectively.

### 2.4. DNA Concentration and Quality

Samples artificially inoculated with *K. pneumoniae* were prepared to compare the DNA extraction protocols. The DNA concentration was determined with a dsDNA Broad Range (BR) kit (Invitrogen^TM^, ThermoFisher Scientific, Waltham, MA, USA), and the measurements were performed in a Qubit fluorimeter (Invitrogen^TM^, ThermoFisher Scientific, Waltham, MA, USA). Regarding the quality, it was determined based on the absorbance ratios of 260/280 and 260/230 provided by a NanoDrop Lite Plus device (Thermo Scientific, Waltham, MA, USA).

### 2.5. khe-LAMP

#### 2.5.1. Real-Time *khe*-LAMP (qLAMP)

The qLAMP assay targeted the *khe* gene; the primer sequence is provided in Table 2. The final reaction volume was 20 μL, which was composed of 12 µL of GspSSD2.0 Isothermal Mastermix (ISO-004, OptiGene, Horsham, UK), 1 μL of 20× primer mix (the final reaction concentrations were 800 nM of FIP/BIP primers, 400 nM of LB/LF primers, and 200 nM of F3/B3), 0.4 μL of ROX, serving as the reference dye (50 nM), 2 μL of template DNA, and 4.6 μL of nuclease-free water. All the samples were analyzed in technical duplicates unless otherwise stated. The amplification temperature was 65 °C, which is the optimum for this master mix, and the reactions were incubated for 30 min, acquiring fluorescence in 30 s intervals. Upon completion of the amplification step, a melt-curve analysis was performed, which consisted of an initial heating at 95 °C for 15 s, cooling down to 80 °C for 20 s, and heating again up to 95 °C at 0.05 °C/s increments with fluorescence acquisition after each increment. As acceptance criteria for considering a sample as positive, both technical replicates had to present positive amplification, with a Tm falling within the calculated average temperature ± its standard deviation. The experiments were performed in a QuantStudio 12 k Flex Real-Time PCR system (Applied Biosystems, Thermo Fisher Scientific, Waltham, MA, USA).

#### 2.5.2. Colorimetric *khe*-LAMP (cLAMP)

The cLAMP assays were performed in 1.5 mL tubes in a dry bath (Thermomixer, Eppendorf AG, Hamburg, Germany), set at 65 °C, and incubated for 30 min. The reactions were prepared with the same composition as the qLAMP described above, but without the addition of the reference dye; thus, 5 μL of nuclease-free water was added to complete the final 20 μL. For color development, SYBR Green I was added. To accomplish this, without interfering with the amplification, once the tubes were loaded with the reaction mix and sample, the top of the tube was covered with Parafilm^®^, leaving an opening of 1–2 mm on the hinge side of the tube. The 1 μL of SYBR Green I 1000× (Invitrogen^TM^, Thermo Fisher Scientific, Waltham, MA, USA) was carefully placed on the center of Parafilm^®^. Lastly, the lid was closed, paying attention to avoid the SYBR from dropping into the reaction, and the tubes were placed in the Thermomixer. Once the incubation was completed, the tubes were vigorously shaken to ensure the mixing of the reaction with the SYBR Green I through the opening left in the Parafilm^®^ and then spun down for 10 s. The positive samples developed a greenish color, while the negative ones remained orange. In addition to this, under UV light, the positive samples emitted fluorescence, while the negative ones did not.

### 2.6. LAMP Validation

#### 2.6.1. Evaluation of Inclusivity and Exclusivity

The inclusivity and exclusivity of the new set of primers was initially assessed in silico by nucleotide BLAST analysis (https://blast.ncbi.nlm.nih.gov/Blast.cgi?PROGRAM=blastn&PAGE_TYPE=BlastSearch&LINK_LOC=blasthome, accessed on 12 March 2025). This was followed by in vitro confirmation against the panel of target and non-target bacteria detailed in Table 1.

#### 2.6.2. Dynamic Range

The dynamic range covered with the newly designed LAMP primers was evaluated after applying the four different protocols described in M&M 2.2 to the three matrixes under study, i.e., milk, blood, and urine, inoculated with decreasing concentrations of WDCM 00192.

#### 2.6.3. Determination of the Limit of Detection (LOD) and Relative Limit of Detection (RLOD)

The limit of detection (LoD), with a confidence of 50% (LOD50) and 95% (LOD95), was determined for the four DNA extraction protocols in the three different matrixes. To this end, the mathematical model described by Wilrich and Wilrich was applied [14]. *K. pneumoniae* WDCM 00192 was spiked into the different matrixes with decreasing concentrations, 1 mL was taken, mixed with 9 mL of Tryptic Soy Broth (TSB, PanReac Applichem, Castellar del Valles, Barcelona, Spain), and enriched for 6 h; then, the extraction protocols were applied and analyzed by qLAMP and cLAMP. This was performed until a spiking concentration with positive and negative results was reached. A total of 20 samples were inoculated, 12 in the range of 10–50 CFU/mL and 8 below 10 CFU/mL; also, one non-spiked sample was included as a negative control. The data were used as input for the model.

For the calculation of the RLOD, a culture-based method was applied as a reference. The same procedure used for the LAMP tests was followed, except that after the 6 h TSB enrichment step, the samples were streaked on MacConkey agar, and the plates were incubated at 37 °C overnight and screened for typical colonies. The determination of the RLOD was performed as indicated in NordVal [15].

### 2.7. Graphical Representation

The data obtained in the present study were represented with GraphPad Prism 10 (GraphPad Software, San Diego, CA, USA, www.graphpad.com).

## 3. Results

### 3.1. LAMP Assays Evaluation

#### 3.1.1. Inclusivity/Exclusivity

The first step to assess the inclusivity/exclusivity of the newly designed primers consisted of in silico analyses performing a BLAST, at the NCBI website. Overall, the top hits returned by the BLAST all corresponded to *K. pneumoniae*.

These analyses were followed by in vitro confirmation by qLAMP. A panel of 12 non-target bacterial species, belonging to 9 different genera, were analyzed. No unspecific amplification was observed with any of these microbes, thus confirming the specificity of the assay.

The specific Tm value for the positive samples was experimentally determined to be 89.4 ± 0.6 °C. The specific combination of the extraction protocol and matrix reported slight differences in the Tm values obtained. In this sense, the values were CH 89.4 ± 0.1 °C, 89.7 ± 0.2 °C, and 89.9 ± 0.1 °C for blood, milk, and urine, respectively.

#### 3.1.2. DNA Extraction Protocol Comparison and *khe*-LAMP Dynamic Range

The dynamic range was evaluated in the three different matrixes after the application of the four extraction/purification protocols.

##### Milk

**Figure 1:**
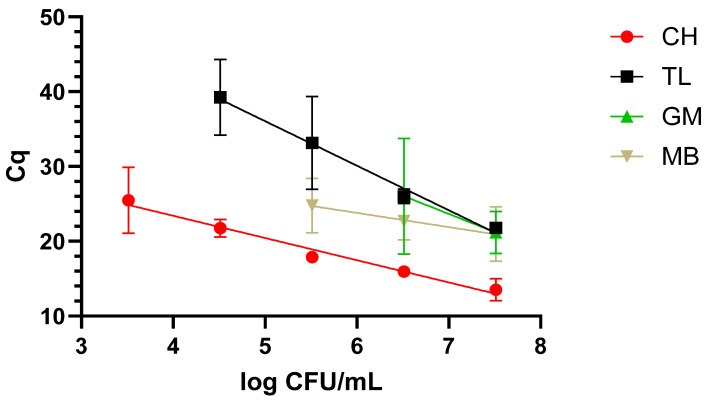
Dynamic range in milk with the four extraction methods: chelex (CH), thermal lysis (TL), glass milk (GM), and magnetic beads (MB). “Cq” stands for cycle of quantification [16].

The protocol returning the best results was the CH one, as it was possible to reach the lowest concentration, 3.5 log CFU/mL, along with a faster amplification over a 5-log range. This was followed by TL, which covered a 4-log range, reaching 4.5 log CFU/mL. The worst performing protocols were MB and GM, covering a range of 3 and 2 logs and reaching 6 and 7 log CFU/mL. These data are graphically represented in Figure 1.

##### Blood

**Figure 2:**
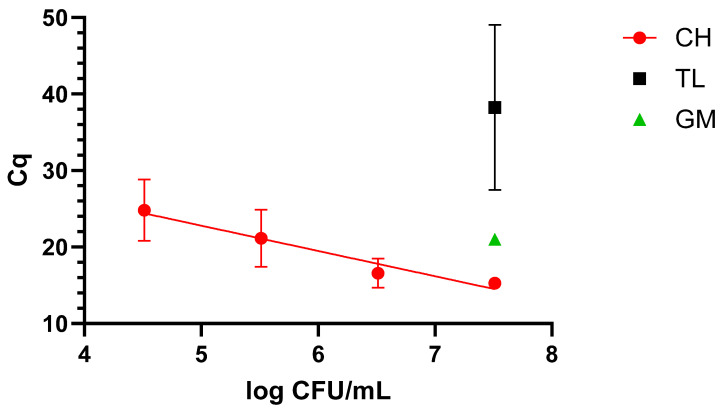
Dynamic range in blood with the four extraction methods: chelex (CH), thermal lysis (TL), and glass milk (GM). “Cq” stands for cycle of quantification [16].

When dealing with blood, only the CH protocol demonstrated suitability for qLAMP application, as it was possible to cover a 4-log range, reaching 4.9 log CFU/mL, as shown in Figure 2.

##### Urine

**Figure 3:**
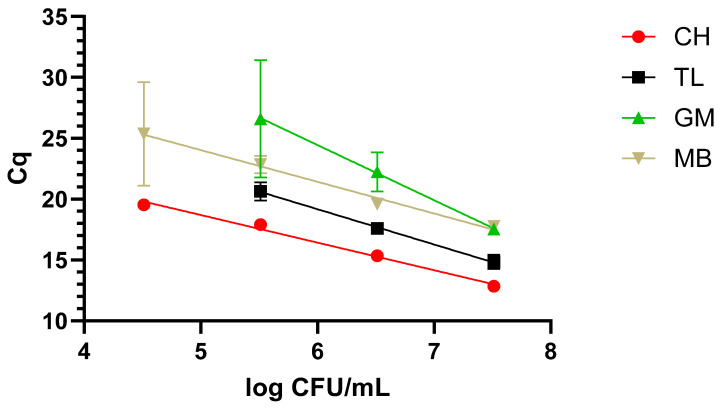
Dynamic range in urine with the four extraction methods: chelex (CH), thermal lysis (TL), glass milk (GM), and magnetic beads (MB). “Cq” stands for Cycle of quantification [16].

When evaluating the dynamic range in the urine samples, a similar trend to that of milk was observed. In this sense, CH performed the best, as it was possible to cover a 4-log range, reaching 5.5 log CFU/mL with the fastest amplification times. This time, the second protocol in terms of performance was the MBs, reaching the same analytical sensitivity as CH but with longer amplification times. Finally, TL and GM obtained the same analytical sensitivity of 6.5 log CFU/mL, in a 3-log range, where TL was faster than GM; see Figure 3.

### 3.2. DNA Extraction Protocol Comparison

When comparing the four different DNA extraction/purification protocols in the three matrixes under study, no major differences were observed in the 260/280 or in the 260/230 absorbance ratios. In the first case, the values ranged from 0.8 to 1.1 in milk, 0.6–1.6 in blood, and 1.5–1.6 in urine. In the later parameter, the values were 0.2–0.3, 0.3–0.4, and 0.2–0.6 for milk, blood, and milk, respectively. See Table 3 for details.

Regardless of the purity values, greater differences were observed when focusing on the concentration of DNA. In this case, the CH protocol obtained the highest concentrations in milk and blood of 918.8 ± 186.3 and 22.5 ± 17.0 ng/µL, respectively, and the second-to-highest concentration in urine of 675.8 ± 117.6 ng/µL, only surpassed by the TL protocol, with 841.7 ± 371.9 ng/µL. The MBs and GM produced the lowest concentrations in all three matrixes. See Table 3 for a summary of the data and Table 4 for the performance of the different protocols with the qLAMP assay.

### 3.3. Determination of the LOD50 and RLOD

**Figure 4:**
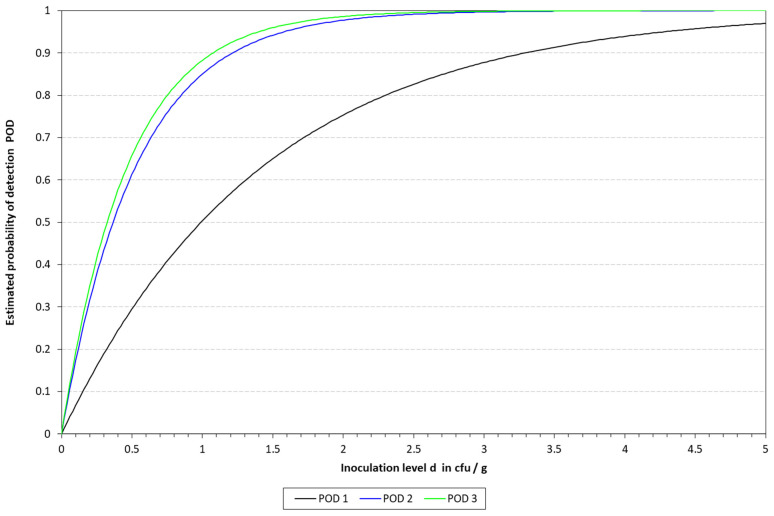
Graphical representation of the LOD calculated for qLAMP with the model described by Wilrich and Wilrich [14]. POD 1, 2, and 3 indicate the probability of detection of *K. pneumoniae* in blood, milk, and urine, respectively.

**Figure 5:**
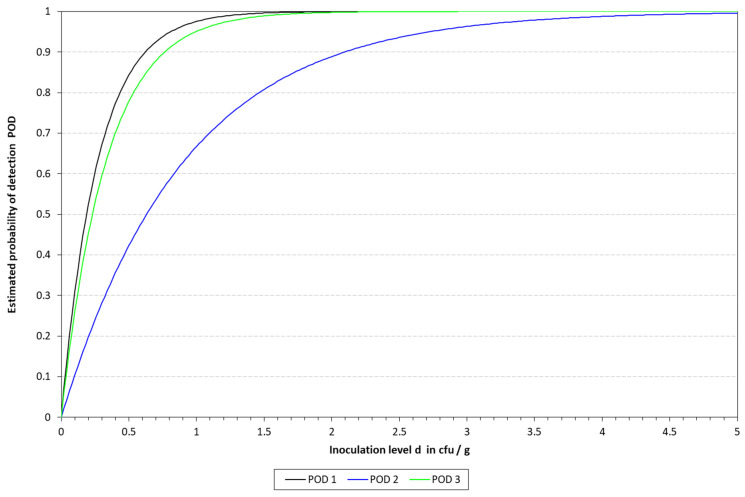
Graphical representation of the LOD calculated for cLAMP with the model described by Wilrich and Wilrich [14]. POD 1, 2, and 3 indicate the probability of detection of *K. pneumoniae* in blood, milk, and urine, respectively.

**Figure 6:**
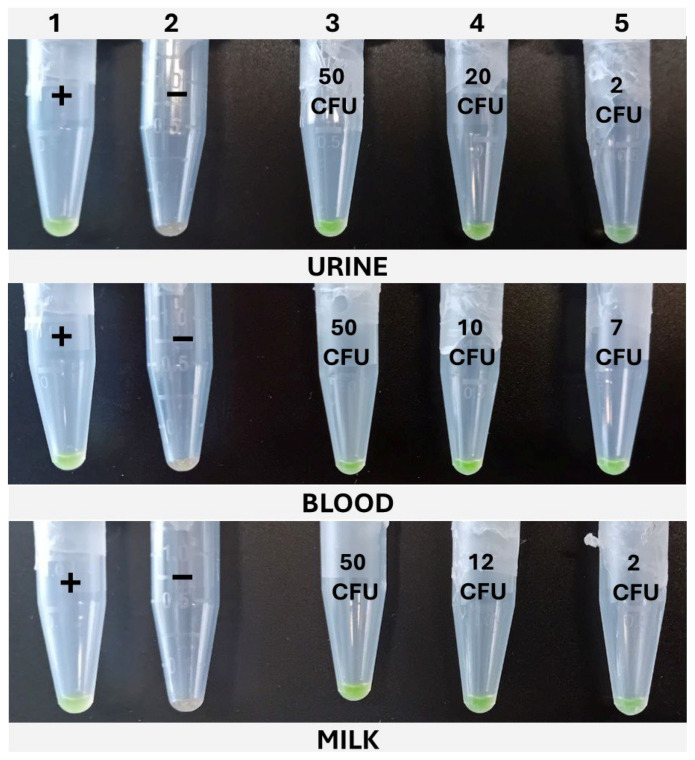
Typical cLAMP results obtained for the three matrixes under study. Column 1, positive control (+). Column 2, negative control (−). Column 3, high inoculation level. Column 4, intermediate inoculation level. Column 5, low inoculation level.

The LOD50 calculated for qLAMP was 9.90, 3.65, and 3.23 CFU/10 mL in blood, milk, and urine, respectively, while it was determined to be 1.87, 6.3, and 2.30 for those matrixes when applying the cLAMP assay. See Table 5 for a summary of the spiked samples, along with the results obtained with the different methods tested. The LOD results calculated are provided in Table 6 and Table 7, along with Figure 4 and Figure 5. When compared to the culture-based approach, the RLOD values were 5.30, 0.58, and 1.40 for qLAMP in blood, milk, and urine and 0.28, 0.66, and 0.62 for cLAMP. The typical colorimetric result is presented in Figure 6.

## 4. Discussion

*K. pneumoniae* is an opportunistic human pathogen of great health concern for its acquisition of antibiotic resistance genes [17]. Typical diagnostic procedures include culturing and isolation of the bacteria from infected individuals or samples.

The newly designed primers specifically amplified DNA from *K. pneumoniae* without unspecific amplification from non-target bacterial species, even from closely related ones such as *E. coli*, thus confirming the in silico results obtained by BLASTn. The use of the hemolysin gene *khe* has already been reported to be suitable for the detection of this pathogen, along with other DNA-based techniques [18,19]. However, not many LAMP-based methods have been reported to target this gene; thus, our study adds to the existing applications.

Typically, when dealing with nucleic-acid-based assays, one or several extraction protocols are compared to determine the optimal one. In this sense, four different protocols were included in our study, but going beyond typical applications, these were compared in three different matrixes, namely, blood and urine, as potential clinical samples, and milk, as a food one. Additionally, not only the final DNA quality and concentration were determined, but also the dynamic range that could be covered with each one of these protocols. As expected, differences were not only observed in regard to the concentration and quality, but the final performance also varied greatly depending on the matrix to which they were applied. In this sense, taken together, the chelex-based protocol provided the best results, as it allowed us to recover amplifiable DNA from all three matrixes, with a dynamic range of 4 to 5 log CFU/mL depending on the matrix under study. It is worth noting that the clinical samples presented the lowest dynamic range with 5 log CFU/mL. The difficulties in the detection of *K. pneumoniae* in clinical samples are most likely associated with the presence of LAMP inhibitors as, even though it has traditionally been claimed that it is more robust than PCR/qPCR [10,20], the technique still presents some degree of susceptibility to certain compounds, which may cause not only a delay in the amplification but also quenching of the fluorescence signal, like hematin in blood [21,22]. In line with this observation is the fact that slight differences in the Tm values recorded for each matrix were observed [23,24]. Among the different matrixes tested, blood was demonstrated to be the most challenging one, as only one out of four protocols was capable of obtaining amplifiable DNA with enough quality and concentration to keep covering a 4-log dynamic range. This protocol was the chelex-based one, which was previously reported to provide good results with this type of sample, and it even obtained results comparable to a commercial kit [25]. In line with this observation, good results were also reported when applied to fecal samples [13], even though Janíková et al. indicated that when dealing with saliva samples, the use of chelex did not improve their capacity to detect SARS-CoV-2 [26]. These discrepancies highlight the importance of accurately evaluating and selecting an appropriate DNA extraction protocol for the sample under study.

Attending to the findings reported above, it was decided to proceed with the chelex protocol to determine the LOD in the spiked samples. The LOD50 reached in all the matrixes was below 10 CFU/mL, thus demonstrating the sensitivity of the final method developed. These results are better than those previously reported by LAMP assays applied to the detection of different microbial pathogens in clinical samples [27,28]. However, it is important to note that the study reported herein includes a short enrichment step, which might be responsible for the higher sensitivity and lower LOD reached by our method. Differences were observed depending on the type of LAMP assay applied for the detection. With qLAMP, similar LOD50 values were calculated for milk and urine, i.e., 3.65 vs. 3.23 CFU/mL, respectively, while when dealing with blood, this value increased up to 9.90 CFU/mL. The limitations mentioned above might be responsible for the differences observed among the three matrixes. On the other hand, when applying the cLAMP assay, the LOD50 values were lower than those obtained by qLAMP when dealing with blood and urine, while they were slightly higher for milk. The better performance of cLAMP compared to qLAMP when analyzing blood might be due to fluorescence quenching, as reported by Nwe et al. [21]. Previous studies have reported that LAMP-based assays for the detection of *K. pneumoniae* provide low LOD values even without the enrichment step; however, it must be kept in mind that these assays rely on more complex DNA extraction protocols, like the study described by Banerjee et al. [29], or additional sample pre-treatment steps, such as immunomagnetic separation, as described by Zhang et al. [30]. Therefore, these variations, in addition to the selection of different genetic targets, may account for the differences reported. The results obtained are in line with those reported for PCR by Khazani et al., who reached log 4 CFU/mL without sample enrichment [31]. In addition to this, it is worth noting that the corresponding LOD values of all these alternative methods were evaluated in other ways than the one reported herein.

The final method developed and presented herein provides key benefits for both the food industry and clinical diagnostics. The real-time detection method is particularly suited for well-equipped laboratories, while the colorimetric method can find a niche in decentralized setups or laboratories with lower resources. This last scenario might be found in in-farm analyses or situations similar to those that occurred during the recent SARS-CoV-2 pandemic. However, caution must be taken as, just like any other molecular and culture-based method, LAMP is not free from assay contamination.

To conclude, the newly designed primers based on the hemolysin gene *khe* allowed for the specific detection of *K. pneumoniae*. The chelex-based DNA extraction/purification protocol was demonstrated to be the best option due to its optimal performance regardless of the type of sample analyzed. The resulting final method reached an LOD50 below 10 CFU/mL regardless of the detection strategy followed, thus providing analytical solutions to a wider variety of laboratories.

## Figures and Tables

**Table 1:** Strains selected for inclusivity and exclusivity studies.

Species	Reference	qLAMP
*K. pneumoniae*	WDCM 00192	+
*Salmonella* Typhimurium	WDCM 00031	−
*Escherichia coli*	WDCM 00013	−
*Acinetobacter baumannii*	CECT 452	−
*Staphylococcus aureus*	WDCM 00035	−
*Staphylococcus epidermidis*	WDCM 00036	−
*Streptococcus agalactiae*	CECT 183	−
*Streptococcus uberis*	CECT 994	−
*Enterococcus faecalis*	WDCM 00009	−
*Listeria monocytogenes*	WDCM 00110	−
*Clostridium difficile*	CECT 531	−
*Clostridium perfringens*	WDCM 00007	−
*Bacillus cereus*	WDCM 00151	−

CECT: Spanish Type Culture Collection. WDCM: World Data Centre for Microorganisms.

**Table 2:** LAMP primers.

Primer Name	Sequence (5′→3′)	Reference
FIP_khe	GAC GAA CTT CCT GCT CGG TGT T *tttt* ACA GCG TGG GTT TTC CCG	This study
BIP_khe	GGC CAA CAA GAA GTA CAA CCG C *tttt* GTC AAC CCA ACG ATC CTG G	
F3_khe	TGG AAG CTG GAG CCC G	
B3_khe	GTG TGG ACC GAA GAA CTG C	
LF_khe	TTG AGA AAG GTG TGG CAG ATG C	
LB_khe	TAC CCG CTC AAT CCC GG	

*tttt* represents a polyT linker between F2 and F1c and B2 and B1c.

**Table 3:** DNA extraction quality and concentration.

Extraction Protocol	Milk			Blood			Urine		
	A260/280	A260/230	[ng/µL]	A260/280	A260/230	[ng/µL]	A260/280	A260/230	[ng/µL]
TL	0.8 ± 0.1	0.2 ± 0.0	126.6 ± 105.7	1.3 ± 0.1	0.3 ± 0.1	13.4 ± 3.4	1.6 ± 0.0	0.3 ± 0.1	841.7 ± 371.9
CH	0.9 ± 0.1	0.2 ± 0.0	918.8 ± 186.3	0.6 ± 0.3	ND	22.5 ± 17.0	1.6 ± 0.0	0.4 ± 0.2	675.8 ± 117.6
MB	0.9 ± 0.0	0.3 ± 0.0	247.5 ± 163.4	1.6 ± 0.1	0.4 ± 0.1	11.2 ± 11.2	1.6 ± 0.8	0.2 ± 0.2	3.2 ± 3.5
GM	1.1 ± 0.2	0.3 ± 0.0	40.3 ± 25.8	1.4 ± 0.3	0.3 ± 0.1	7.8 ± 4.1	1.5 ± 0.1	0.6 ± 0.1	15.2 ± 4.2

ND: Not Determined.

**Table 4:** DNA extraction comparison by qLAMP.

log CFU/mL	Milk				Blood				Urine			
	TL	CH	MBs	GM	TL	CH	MBs	GM	TL	CH	MBs	GM
8.5–7.5	21.8 ± 0.5	13.5 ± 1.5	20.9 ± 3.6	21.2 ± 2.8	38.2 ± 10.8	15.3 ± 0.8	ND	21.0 ± 0.3	15.2 ± 0.2	12.8 ± 0.1	17.8 ± 0.4	17.6 ± 0.4
7.4–6.5	26.0 ± 0.9	15.9 ± 0.1	22.8 ± 2.6	26.1 ± 7.8	ND	16.6 ± 1.9	ND	ND	17.6 ± 0.1	15.3 ± 0.2	19.6 ± 0.4	22.2 ± 1.6
6.4–5.5	33.2 ± 6.2	17.9 ± 0.3	24.8 ± 3.7	ND	ND	21.1 ± 3.8	ND	ND	20.6 ± 0.8	17.9 ± 0.4	22.8 ± 0.7	26.6 ± 4.8
5.4–4.5	39 ± 5.1	21.8 ± 1.2	ND	ND	ND	21.8 ± 1.3	ND	ND	ND	19.6 ± 0.3	25.4 ± 4.3	ND
3.5	ND	25.5 ± 4.4	ND	ND	ND	ND	ND	ND	ND	ND	ND	ND
2.5	ND	ND	ND	ND	ND	ND	ND	ND	ND	ND	ND	ND

TL: thermal lysis. CH: chelex. MBs: magnetic beads. GM: glass milk. ND: Not Determined.

**Table 5:** Inoculation results for the determination of the LOD.

CFU/mL	Blood			Milk			Urine		
	qLAMP	cLAMP	MCA	qLAMP	cLAMP	MCA	qLAMP	cLAMP	MCA
50	+	+	+	+	+	+	+	+	+
45	+	+	+	+	+	+	+	+	+
40	−	+	+	+	+	+	+	+	+
35	−	+	−	+	−	+	+	+	+
30	+	+	+	+	+	+	+	+	+
25	+	+	+	+	+	+	+	+	+
20	+	+	+	+	+	+	+	+	+
15	+	+	−	+	+	+	+	+	+
15	+	+	+	+	+	−	+	+	+
12	+	+	+	+	+	−	+	+	+
10	+	+	+	+	+	+	+	+	+
10	−	+	+	−	−	−	+	+	+
9	+	+	+	+	+	+	+	+	−
8	+	+	+	−	−	−	+	+	+
7	−	+	+	+	+	−	−	+	−
5	−	−	−	+	+	+	−	−	−
5	+	+	−	+	+	−	+	+	+
2	−	+	+	+	+	−	−	−	+
2	+	+	+	−	−	−	+	+	−
1	−	−	−	−	+	−	−	−	+
0	−	−	−	−	−	−	−	−	−

MCA: MacConkey Agar. cLAMP: colorimetric LAMP. qLAMP: real-time LAMP.

**Table 6:** LOD comparison among different matrixes with qLAMP.

Matrix	LOD50			LOD95		
	Detection Limit	Lower Conf. Limit	Upper Conf. Limit	Detection Limit	Lower Conf. Limit	Upper Conf. Limit
Blood	9.90	5.03	19.50	42.78	21.72	84.27
Milk	3.65	1.76	7.59	15.78	7.59	32.81
Urine	3.23	1.53	6.83	13.97	6.61	29.53

LOD50: limit of detection 50%, LOD95: limit of detection 95%.

**Table 7:** LOD comparison among different matrixes with cLAMP.

Matrix	LOD50			LOD95		
	Detection Limit	Lower Conf. Limit	Upper Conf. Limit	Detection Limit	Lower Conf. Limit	Upper Conf. Limit
Blood	1.87	0.78	4.50	8.08	3.56	19.45
Milk	6.30	3.17	12.52	27.24	13.72	54.09
Urine	2.30	1.02	5.20	9.94	4.40	22.46

LOD50: limit of detection 50%, LOD95: limit of detection 95%.

## Data Availability

The original contributions presented in this study are included in the article; further inquiries can be directed to the corresponding authors.
